# Will Investments in Large-Scale Prospective Cohorts and Biobanks Limit Our Ability to Discover Weaker, Less Common Genetic and Environmental Contributors to Complex Diseases?

**DOI:** 10.1289/ehp.7343

**Published:** 2004-11-04

**Authors:** Morris W. Foster, Richard R. Sharp

**Affiliations:** ^1^Department of Anthropology, University of Oklahoma, Norman, Oklahoma, USA; ^2^Center for Medical Ethics and Health Policy, Baylor College of Medicine, Houston, Texas, USA

**Keywords:** biobanks, communities, complex disease, gene–environment interaction, prospective cohorts, qualitative methods, research design

## Abstract

Increasing the size of prospective cohorts and biobanks is one approach to discovering previously unknown contributors to complex diseases, but it may come at the price of concealing contributors that are less common across all the participants in those larger studies and of limiting hypothesis generation. Prospective cohorts and biobanks constitute significant, long-term investments in research infrastructure that will have ongoing consequences for opportunities in biomedical research for the foreseeable future. Thus, it is important to think about how these major additions to research infrastructure can be designed to be more productive in generating hypotheses for novel environmental contributors to complex diseases and to help identify genetic and environmental contributors that may not be common across the larger samples but are more frequent within local or ancestral subsets. Incorporating open-ended inquiries and qualitative information about local communal and ecologic contexts and the political, economic, and other social structures that affect health status and outcome will enable qualitative hypothesis generation in those localized contexts, as well as the collection of more detailed genealogic and family health history information that may be useful in designing future studies. Using communities as building blocks for larger cohorts and biobanks presents some practical and ethical challenges but also enhances opportunities for interdisciplinary, multilevel investigations of the multifactorial contributors to complex diseases.

Of the approximately 30,000 genes in the entire human genome, > 1,500 genetic variants have been discovered in which a single allele (either as a homozygote or heterozygote) is sufficient for a single gene or Mendelian disorder such as Huntington’s disease to develop (National Center for Biotechnology Information 2004). However, relatively few variants have been confirmed for complex diseases such as cancer, heart disease, and diabetes in which both susceptibility genes and environmental contributors are required for the disease to develop (Botstein and Risch 2003; Hirschhorn et al. 2002). The slow pace in identifying and confirming genetic contributors for complex diseases is due primarily to the difficulties of detecting relatively weak, incremental genetic effects as well as to the possibility that even moderate or strong effects involving a genetic contributor may require the co-occurrence of one or more environmental contributors (Hodgson and Popat 2003).

Similarly, although the identity and function of some environmental contributors to complex diseases such as cancer are well known (toxicants such as asbestos, behaviors such as smoking, viruses such as human papilloma virus), almost all of these known contributors have been identified as such because they have relatively strong effects on disease susceptibility. At the same time, however, a significant proportion of environmental contributors remain unknown for many complex diseases. For example, only one-third of the breast cancer cases in the United States can be accounted for by known risk factors (Stevens 2002). The overwhelming remainder involves either candidate risk factors that are known but have not yet been confirmed as such (which raises the cases accountable to ~50%) or risk factors that are not recognized as such at all. Moreover, even already-identified risk factors for disease such as diet, tobacco, and hormones each are composed of complicated combinations of behaviors and toxicants whose roles in carcinogenesis are not well understood (Brennan 2002). Smoking, for instance, is a contextually shaped behavior that can take a variety of often culturally specific forms as it exposes those who perform it (and others around them) to > 300 different toxicants (Chassin et al. 2000; Frohlich et al. 2002).

In response to these current limitations, a number of researchers have suggested scaling up research sample sizes to provide greater statistical power for identifying and confirming genetic and environmental contributors to complex diseases (Caporaso 2002; Collins 2004; Little et al. 2003; Millikan 2002). Efforts in scaling up sample sizes involve significant national and private investments in research infrastructure. Governmental and nonprofit funding agencies as well as for-profit ventures in various countries are in the process of planning or assembling larger scientific resources to meet that perceived need.

Some of these larger sample collections are in the form of prospective cohorts that recruit healthy participants with the intention of following their health status over a number of years. For example, the National Institute of Child Health and Human Development along with the National Institute of Environmental Health Sciences, the Centers for Disease Control and Prevention, and the U.S. Environmental Protection Agency has been planning a National Children’s Study designed to follow 100,000 children and their parents over multiple decades (National Children’s Study 2004), and the National Cancer Institute (NCI) has recently issued a new call for proposals for funding large prospective cohorts (NCI 2003). The NCI already funds the Black Women’s Cohort (64,500 participants) and the California Teachers Study (133,479 participants) among other large prospective studies (NCI 2004). The NCI announcements of funding for prospective cohorts explicitly contrast them with previous investments in cross-sectional or case–control studies, characterizing cohorts as more flexible, longer-lasting investments in research infrastructure. Most recently, the National Human Genome Research Institute, in collaboration with the National Heart, Lung, and Blood Institute, has requested information from researchers in planning a national cohort of 500,000 participants (National Institutes of Health 2004). In Europe, there is a long tradition of birth cohort studies that extend decades into adulthood, with recent investments in new birth cohorts by the United Kingdom and planning for a “mega” cohort by the European Union (Kogevinas 2002).

A related kind of resource, often called biobanks, incorporates members of national or regional populations for which extensive retrospective medical records, DNA samples, and other health-related information are available to researchers (Austin et al. 2003). Some biobanks also function as prospective cohorts. The deCODE project, for instance, already has isolated genes that appear to contribute to osteoporosis, stroke, diabetes, and several other complex diseases using historical and contemporary health information and DNA samples from more than 100,000 residents of Iceland (deCODE Genetics 2004), although some of those findings may turn out to be limited to rarer familial factors. Similar biobanks are being assembled in Estonia (an open-ended number of participants), the United Kingdom (500,000 participants), Quebec (60,000 participants), and Japan (300,000 participants). In the United States, Howard University has announced the formation of a biobank with samples from participants who identify themselves as African Americans (Kaiser 2003).

Investments made today in prospective cohorts and biobanks that are projected to be used (and funded) for decades to come will have significant consequences for determining both the opportunities and the limits of future research into genetic and environmental contributors to complex diseases. Although it will be possible to establish new cohorts and biobanks in the future, it will be several years before prospectively recruited participants develop diseases of interest in sufficient numbers for analysis. Moreover, funding for additional cohorts in future years will compete with the costs of maintaining ongoing cohorts, which likely will limit future growth in this research infrastructure. Consequently, as cohorts and biobanks are being planned, it is important to consider the methodologic implications that their increased scales may have for identifying genetic and environmental contributors that may be more locally variable in effect. Locally variable or less common contributors nonetheless can have significant effects on health disparities, raising questions about the equitable distribution of research benefits in the case of large, expensive cohorts that may not be designed to attend to smaller-scale contexts.

## Is Bigger Always Better?

Although larger cohorts or biobanks likely will help identify many genetic and environmental contributors that are more common among their members, they will be less likely to help identify those less common contributors that are rare among most participants. Indeed, the additional power that a larger cohort provides to detect weaker common effects simultaneously can mask those contributors that are localized primarily within subsets of the larger sample, depending on how cohort information is collected and analyzed. For instance, a genetic variant that is more frequent among individuals of a particular ancestry but rare among others may not be detected in a sample of 100,000 participants recruited using such inclusion criteria as regional or national residence or occupation. Similarly, an environmental contributor that is specific to exposures resulting from a local ecologic feature or a locally specific behavior also could be lost in a large, multisite cohort, even though it may be a significant determinant of disease. This means that the ways in which participants are categorized and recruited for a particular cohort or biobank and in which their information is collected and analyzed will affect what studies using that resource may find as well as what they may miss.

A criticism of the UK Biobank, for instance, has been that it has no specific plans to incorporate a familial component into its recruitment strategy (Wright et al. 2002). Family members (particularly sibling pairs and parents) provide greater power for separating genetic effects from the background noise of nongenetic effects. In addition, there also tend to be correlations in common environmental and gene–environment interactions among close relatives compared with random, unrelated individuals. Thus, the larger size of a cohort may not necessarily increase its power to detect genetic or environmental contributors to complex diseases.

That situation is complicated further by the possibility that the same complex disease may have multiple genetic and environmental contributors that are neither necessary nor sufficient for a similar phenotype to be expressed (Smith and Lusis 2002). In the cases of type 2 diabetes and systemic lupus erythematosus, for example, different candidate genes have been proposed from studies of geographically and ancestrally differing patient populations, although some of those will not be confirmed (Kelly et al. 2002; Stern 2002). In the case of breast cancer (as for the vast majority of other cancers), not all confirmed environmental contributors need be present for the disease to develop. With the additional variable of gene–environment interactions, it may well be that some significant (although still minority) proportions of the incidence of most complex diseases are attributable to intersections of locally varying combinations of genetic and environmental contributors some or even many of which may not be detectable in large multisite samples. To the extent that those polygenic and polyenvironmental contributors are nonrandomly distributed among and across populations, a large cohort or biobank may fail to detect some or even most of these unless it is structured to support more intensive study of subsets of participants.

The greater cost of larger cohorts, however, tends to mean that fewer and often less precise measures are obtained for each participant, a situation that actually can reduce the power of a larger sample (Wong et al. 2003). Sampling costs also can reduce the ability to collect information that is most productive for hypothesis generation. Because it is expensive to investigate family histories and environmental exposure histories for large numbers of participants (Barbour 2003), large cohort studies tend to collect participant information through closed-ended questions—that is, by giving participants a range of predetermined answers to predetermined questions and forcing them to choose among them (UK Biobank 2002). For environmental exposures, closed-ended questions are useful in testing hypotheses about established or suspected contributors but are of limited value in identifying previously unsuspected contributors whether those are localized or more common (Foster and Aston 2003). With respect to ancestry, some studies allow participants to indicate more than one ethnic or racial background but without eliciting additional information that may be more informative about how genetic variants are distributed in the extensive middle ground between immediate family members and large population categories such as European American or African American.

These limited, closed-ended responses frequently are used as proxies for a shared population history (in the case of ancestry) or for shared environmental exposures (or both) for purposes of sample stratification. The difficulty, however, is that such broad, decontextualed proxies often are treated as units of analysis rather than as heuristic means to disambiguate or discover specific ancestral and environmental contributors to disease or to provide a degree of diversity within the sample frame.

Identity alone, however, is not causal and may not even necessarily be predictive. First, not all factors linked to a given identity necessarily contribute to disease expression or to the expression of the same diseases. Second, only some environmental and ancestral factors are shared among those with a common identity. Third, only some of those with a common identity necessarily share those linked factors. Social identity does become a more powerful predictor, however, when it intersects with ecology in a specific locality. Sharing both a social identity and a locality increases the likelihood that and the extent to which a social community will regulate the actions of its members according to some standard of appropriateness (and, hence, manifest many of the same behavioral environmental factors), the likelihood that community members are exposed to many of the same ambient factors in the physical environment, and the degree of access to prevention, surveillance, and treatment available to community members. Locality also may limit significantly the number of ancestries shared among co-residents while increasing the likelihood that some are related by more immediate genealogic connections.

## Communities as Building Blocks

These critiques suggest that a significant challenge in constructing large-scale cohorts and biobanks is to design a study with a large number of participants that nonetheless gathers rich data on individuals and the contexts that affect their health, providing flexibility for discovering unanticipated data fields and new categories within existing fields. One solution may be to use the local communities in which individuals are everyday members—a naturally occurring social middle ground between single participants and very large ethnic and other categories—as building blocks for constructing large prospective samples. Local communities also would be appropriate contexts for recruiting parents and siblings to enrich the familial component of cohorts and biobanks.

Local communities, of course, may be quite variable in form, ranging from relatively well-defined residential clusters or towns in rural areas to neighborhoods or social networks within large metropolitan areas. What defines a localized community, however, is that its members share similar interactional conventions, a consequence of their everyday encounters with one another, as well as similar ambient or background exposures due to the local physical environment.

The idea that locality or place may affect health is not new (Durkheim 1951). However, the last decade has seen a revival of interest in theorizing and conceptualizing that relationship (Curtis and Rees-Jones 1998; Kearns and Joseph 1993; Macintyre et al. 1993; Tunstall et al. 2004). In contrast with the prevailing epidemiologic focus on individual risk factors, this revival has emphasized collective or contextual effects that may mediate the effects of individual-level variables such that the health status of individuals depends to some extent on the social and physical environments in which individuals grow up and live (Schwarz 1994; Susser 1994). The proponents of this approach argue that collective or contextual “area effects” are complex, multilevel interactions involving phenomena or forces ranging from global, national, or regional social structures that determine opportunities and limitations for well-being (including economic systems and conditions, health care systems and access, political structures and equity, and widespread cultural beliefs and social practices) to more localized communal beliefs, practices, and conditions to diverse intracommunity patterns of individual agency (Macintyre et al. 2002; Popay et al. 1998). Thus, rather than adopt the traditional epidemiologic practice of isolating and testing one environmental factor at a time while attempting to control for the effects of others, a more appropriate method of analysis may be to embrace the complexity of multilevel collective or contextual contributors.

Fine-grained information about contextual effects in local communities offers two primary advantages in studies of environmental contributors to disease susceptibility. First, those data provide additional background information that can be used to better interpret responses to standardized questions, but in ways that still allow comparison across the larger sample. For instance, the same ethnic identity or household income level can indicate differing health risks and outcomes depending on such locally variable contributors as beliefs about health and illness, familial and communal social dynamics and networks, and political and economic structures (Krieger 2001; Williams 2003). Each of these parameters (along with others) helps shape everyday life in ways that can have differing consequences for behaviors that may expose individuals to environmental toxins and may be further differentiated by local variations in physical environments and the ambient exposures that those offer. Detailed investigations of these local differences can augment an understanding of the pathways by which social and ecologic factors contribute to disease susceptibility or can explain why a risk factor does not appear to be as predictive for a specific sub-population (Frohlich et al. 2001).

Second, detailed local investigations allow many more opportunities for hypothesis generation, which then can be tested across the larger sample. Epidemiologic tests for the statistical significance of associations between established proxies such as ethnicity or socioeconomic status and disease incidence or mortality offer few opportunities for generating novel hypotheses about environmental contributors, mainly because those proxies summarize rather than disaggregate specific environmental factors. In contrast to proxies that summarize information, a community-specific approach that produces large amounts of in-depth information about a broad range of aspects of everyday life provides many specific possibilities for generating hypotheses (Brown 2003; Thompson and Gifford 2000). Indeed, generating hypotheses in small-scale contexts is preferable to doing so across large multisite samples because the former is more amenable to qualitative studies of the different ways in which a large number of factors interact with one another, whereas the latter is more suited to testing hypotheses about a limited number of well-defined, measurable data points.

One of the primary difficulties in using large samples to detect gene–environment interactions is that most nongenetic influences are difficult to measure such that they often are dismissed as being beyond investigation in large samples (Wright et al. 2002). Rather than simply ignore those influences in a larger sample because they cannot be measured accurately or efficiently using existing metrics, qualitative, community-specific approaches offer the possibility of developing a functional understanding of how their effects are achieved, which may help develop accurate, efficient measures that then can be applied in quantitative analyses of larger samples. For example, qualitative data gathered using a “life course” approach can be analyzed to identify biologic and social factors that affect health throughout life in a cumulative manner (both independently and interactively), develop measures of their effects, and describe chains or pathways of risk by which linked exposures raise the likelihood of disease expression (Ben-Shlomo and Kuh 2002; Hallqvist et al. 2004; Kuh and Ben-Shlomo 1997; Kuh et al. 2003). Thus, qualitative methods such as ethnography may become an interdisciplinary companion to epidemiology (Kaufman and Cooper 2001; O’Campo 2003).

## Practical and Ethical Challenges

Taking a community-specific approach does raise several logistical and ethical issues that may be problematic. An immediate reaction to our proposal is likely to be concern about the additional cost of recruiting participants to comprise both local community units and the overall cohort, as well as the additional cost of in-person elicitation of open-ended ethnographic and genealogic information. However, given the significant investments already required by very large prospective cohorts or retrospective biobanks, incurring additional costs to enrich the information collected, particularly with respect to hypothesis generation, should be seen as enhancing the value of what will become long-term investments in biomedical infrastructure. A less expensive alternative could be to recruit some but not all participants as members of community units, with the idea that hypothesis generation need not involve all cohort or biobank participants. Indeed, community units may be selected within the larger scale of the study as a whole in two ways: as models that are representative of most study participants (and so have a likelihood of generating hypotheses that may be tested quantitatively across most participants to identify more common contributors) or as efforts to make the cohort more diverse by including participants whose identities contain elements (e.g., ancestry, residence, occupation, household income) that may evidence some contributors to disease differing from those that are more common within the larger cohort. Both strategies add value to the cohort as a whole, albeit in different ways.

With respect to the latter strategy, a frequent problem in making a participant pool more diverse is that including subjects who may represent minority experiences of disease does not necessarily ensure sufficient power to stratify the sample to quantitatively analyze the less common contributors that may affect those more diverse participants. However, by recruiting some of those minority participants as members of community units, they can be oversampled by the greater detail of information collected rather than by attempting to recruit larger numbers of participants who fit those less common inclusion criteria.

A community-specific strategy also presents several ethical challenges. For example, investigators will need to consider when the additional subject interactions become overly burdensome on particular populations—for example, minority communities that may have been studied extensively in the past. Collecting large amounts of in-depth data about participants, family members, and local communities also presents somewhat greater ethical challenges than do responses to closed-ended questions. Although maintaining confidentiality is a requirement in both cases, it is more difficult to anticipate the risks that might accrue from open-ended inquiries. Moreover, gathering additional information about communities as wholes and about third-party relatives may entail the potential for risks to others than just study participants. For example, published indications of greater genetic susceptibility to a disease among individuals of a specific ancestry or of greater environmental risks to those who reside in a particular place or pursue a particular lifestyle may put those with that ancestry, residence, or lifestyle at a greater risk for discrimination or stigmatization.

At the same time, community-specific investigation often creates a stronger relationship between researchers and participants that should tend to produce greater trust and, hence, more extensive and accurate responses as well as reduced attrition in multiyear and multidecade studies. Emphasizing communities makes it possible to engage pre-existing social organizations and networks in evaluating (and possibly modifying) ethical protections and recruitment strategies, in assisting in participant recruitment and liaison, in actually collecting some study information, and in helping construct local interpretations of the information collected (Sharp and Foster 2000). This greater attention to local contexts should result in greater participant influence in shaping how research is done and greater investigator awareness of local community needs.

## Conclusion

The future of biomedical research should reside both in “small science” and in “big science.” The two approaches are not necessarily mutually exclusive, although the larger scale of the latter may limit the scale of information that is collected from participants. We believe that larger cohorts and biobanks need not preclude smaller, finer-grained investigations of community-specific influences on disease. In fact, qualitative, community-specific investigations are not only possible within the context of those increasingly large-scale investigations but can provide opportunities for additional hypothesis generation as well as facilitate the multilevel analysis of individual, contextual, and structural factors that contribute to complex diseases.
